# Impaired cardiac function is associated with mortality in patients with acute COVID-19 infection

**DOI:** 10.1007/s00392-020-01683-0

**Published:** 2020-06-14

**Authors:** Dominik Rath, Álvaro Petersen-Uribe, Alban Avdiu, Katja Witzel, Philippa Jaeger, Monika Zdanyte, David Heinzmann, Elli Tavlaki, Karin Müller, Meinrad Paul Gawaz

**Affiliations:** Department of Cardiology and Angiology, University Hospital Tübingen, Eberhard Karls Universität Tübingen, Otfried-Müller Str.10, 72076 Tübingen, Germany

**Keywords:** COVID-19, Cardiac function, Prognosis, Cardiovascular disease, Heart failure

## Abstract

**Background:**

COVID-19 infection may cause severe respiratory distress and is associated with increased morbidity and mortality. Impaired cardiac function and/or pre-existing cardiovascular disease may be associated with poor prognosis. In the present study, we report a comprehensive cardiovascular characterization in the first consecutive collective of patients that was admitted and treated at the University Hospital of Tübingen, Germany.

**Methods:**

123 consecutive patients with COVID-19 were included. Routine blood sampling, transthoracic echocardiography and electrocardiography were performed at hospital admission.

**Results:**

We found that impaired left-ventricular and right-ventricular function as well as tricuspid regurgitation > grade 1 were significantly associated with higher mortality. Furthermore, elevated levels of myocardial distress markers (troponin-I and NT pro-BNP) were associated with poor prognosis in this patient collective.

**Conclusion:**

Impaired cardiac function is associated with poor prognosis in COVID-19 positive patients. Consequently, treatment of these patients should include careful guideline-conform cardiovascular evaluation and treatment. Thus, formation of a competent Cardio-COVID-19 team may represent a major clinical measure to optimize therapy of cardiovascular patients during this pandemic.

## Introduction

Coronavirus disease 2019 (COVID-19) may cause severe respiratory distress and has been associated with enhanced morbidity and mortality [1]. There is increasing evidence that COVID-19 may induce severe acute cardiac injury with alterations of cardiac function and elevation of myocardial injury markers including troponin and brain-natriuretic peptide (BNP) [2–4]. Severe compromise of cardiac function and/or pre-existing cardiovascular disease have been reported to be associated with poor prognosis [5, 6]. Thus, the European Society of Cardiology and the American College of Cardiology strongly recommend a careful cardiologic assessment of patients who present with symptomatic COVID-19 infection [7, 8]. Electrocardiographic, echocardiographic and cardiac-specific laboratory parameters are cornerstones to define cardiac injury during infectious disease including COVID-19 [9–11]. In the present study, we report the cardiac-specific findings in the first collective of patients admitted and treated at our university hospital.

## Methods

### Study design and participants

For this consecutive, prospective study, patients were enrolled from February to March 2020 and routine blood samples were collected at hospital admission. We performed transthoracic echocardiography as well as electrocardiography (ECG) within 24 h after admission. Written informed consent was obtained from all non-ventilated patients. Patients were admitted to the University Hospital of Tübingen, Germany. We included 123 consecutive patients diagnosed with severe acute respiratory syndrome coronavirus 2 (SARS-CoV-2). COVID-19 was diagnosed via RNA detection from nasopharyngeal secretions with real-time reverse transcriptase polymerase chain reaction. This study was approved by the institutional ethics committee (238/2018BO2) and complies with the declaration of Helsinki and the good clinical practice guidelines [12–14].

### Transthoracic echocardiography

Transthoracic echocardiography (TTE) was performed in patients with symptomatic and verified COVID-19 infection within 24 h after hospital admission. Left ventricular ejection fraction (LVEF), right ventricular function (RV-function), valve defects as well as pericardial effusion (PE) were evaluated. LVEF was determined using Simpson’s biplane method of discs by manual planimetry of the endocardial border in end-diastolic and end-systolic frames as well as by visual assessment [15]. Impaired LVEF was defined as a systolic ejection fraction ≤ 50% [16]. RV-function was determined by visual assessment, measurement of tricuspid annular plane systolic excursion (TAPSE), and right ventricular fractional area change (RV-FAC). TAPSE was assessed by placing an M-mode cursor through the lateral tricuspid valve annulus in the apical four-chamber view and measuring the total systolic excursion distance of the tricuspid annulus. TAPSE ≥ 20 mm was considered as an indicator for normal RV-function [16]. Right ventricular dysfunction was considered present when RV-FAC was < 35% [16]. Mitral regurgitation was determined by left atrium jet area and width of vena contracta [17]. Aortic stenosis was measured via continuity equitation and planimetry of valve area [18]. Aortic valve regurgitation was determined using jet/left ventricular outflow tract (LVOT) width, diastolic flow reversal in proximal descending aorta as well as pressure half time (PHT) [19]. Finally, tricuspid regurgitation was defined using central jet area and width of vena contracta [17]. When possible, the pulmonary artery pressure was estimated by determining the flow velocity when a tricuspid regurgitation was present [20].

### 12-Channel ECG and laboratory parameters

12-Channel ECG was registered according to standard procedure. Peripheral venous blood was analysed for laboratory parameters including C-reactive protein, troponin I, NT pro-BNP, and d-dimers.

### Clinical follow-up

30-Day follow-up was available for all patients after study inclusion for the primary endpoint all-cause death.

### Statistical analysis

All statistical analyses were performed using SPSS version 26.0 (SPSS Inc., Chicago IL, USA). Normally distributed data were compared using Student’s *T* test. Non-normally distributed data were compared performing Mann–Whitney *U* Test. Mean values are presented as mean ± standard deviation. Median values are presented as median and 25th/75th percentiles. Cross-tabulations with Chi-square tests were performed descriptively to show the number of endpoint distribution. For censored data, Kaplan–Meier curves with log rank tests were determined. Multiple Cox-regression analysis was applied to analyse associations of impaired myocardial function with the endpoint mortality after adjustment for epidemiological factors.

## Results

Baseline characteristics are presented in Tables 1 and 2. Table 1 shows baseline characteristics for the overall collective (*n* = 123) whereas baseline characteristics in Table 2 are stratified according to mortality. We were able to provide echocardiographic data for 98 patients (79.7%), electrocardiographic data for 115 patients (93.5%) and laboratory parameters for all COVID-19 patients.

**Table 1:** Baseline characteristics of the overall cohort (*n* = 123)

Age, years (mean ± SD)	68 (± 15)
Male, *n* (%)	77 (62.6)
Body mass index (mean ± SD)	28 (± 5)
**Cardiovascular risk factors,** ***n *(%)**	
Arterial hypertension	86 (69.9)
Dyslipidemia	46 (37.4)
Diabetes mellitus	30 (24.4)
Current smokers	1 (0.8)
Obesity	24 (19.5)
Atrial fibrillation	28 (22.8)
Known CAD	28 (22.8)
Chronic kidney disease	14 (11.4)
**Echocardiography**	
Left ventricular function, %, mean (± SD)	57 (8)
Left ventricular hypertrophy, *n* (%)	69 (74.2)
Visually estimated normal right ventricular function, *n* (%)	81 (86.2)
Visually estimated impaired right ventricular function, *n* (%)	13 (13.7)
Right ventricular dilatation, *n* (%)	46 (48.9)
TAPSE, mm, mean (± SD)	22 (± 5)
TAPSE < 20 mm, *n* (%)	17 (17.3)
RV-FAC (%)	37 (± 8.9)
RV pressure, mmHg, mean (± SD)	29 (± 11)
Aortic stenosis > 1, *n* (%)	4 (5.7)
Aortic regurgitation > 1, *n* (%)	10 (11.5)
Mitral regurgitation > 1, *n* (%)	24 (26.7)
Tricuspid regurgitation > 1, *n* (%)	30 (30.6)
Pericardial effusion, *n* (%)	45 (48.9)
**Electrocardiography**	
Rate, bpm, mean (± SD)	85 (± 23)
Sinus rhythm, *n* (%)	80 (69.6)
QRS, ms, mean (± SD)	94 (± 22)
Regular R progression, *n* (%)	54 (47)
Right bundle branch block, *n* (%)	4 (3.5)
Left bundle branch block, *n* (%)	1 (0.9)
PQ segment, ms, mean (± SD)	172 (± 94)
QTc, ms, mean (± SD)	445 (± 33)
Negative *T* wave, *n* (%)	13 (11.3)
ST segment depression, *n* (%)	1 (0.9)
ST segment elevation, *n* (%)	0 (0.0)
**Laboratory values at admission, median (25th/75th percentile)**	
Leucocytes, 1000/µl	6.6 (4.4/9.2)
Lymphocytes, 1000/µl	0.8 (0.6/1.1)
Creatinin, mg/dl	0.9 (0.7/1.3)
GFR, ml/m^2^	74 (49/91)
D-dimers, µg/dl	1.2 (0.7/2.8)
C-reactive protein, mg/dl	8.1 (2.6/15.5)
Procalcitonin, ng/ml	0.1 (0.1/0.7)
Troponin I, ng/dl	16 (5/33)
NT pro-BNP, ng/l	445 (139/2714)
CK, U/l	152 (76/320)
AST, U/l	43 (27/63)
ALT, U/l	34 (21/49)
LDH, U/l	334 (242/437)
**Medication at admission,** ***n *(%)**	
Oral anticoagulation	15 (12.2)
ACEi/ARB	60 (48.8)
Aldosterone inhibitors	15 (12.2)
Diuretics	40 (32.5)
Calcium channel blockers	26 (21.1)
Beta blockers	43 (35.0)
Statins	41 (33.3)
ASA	27 (22.0)
P2Y12 inhibitors	3 (2.4)

**Table 2:** Baseline characteristics stratified according to mortality

	Non-survivors	Survivors	*p* value
	(*n* = 16)	(*n* = 107)	
Age, years (mean ± SD)	73 (± 16)	67 (± 15)	0.235
Male, *n* (%)	12 (75.0)	65 (60.7)	0.272
Body mass index (mean ± SD)	30 (± 5)	28 (± 5)	0.183
**Cardiovascular risk factors,** ***n* (%)**			
Arterial hypertension	12 (75.0)	74 (69.2)	0.635
Dyslipidemia	3 (18.8)	43 (40.2)	0.098
Diabetes mellitus	5 (31.3)	25 (23.4)	0.743
Current smokers	0 (0.0)	1 (0.9)	0.707
Obesity	2 (12.5)	22 (20.6)	0.549
trial fibrillation	4 (25.0)	24 (22.4)	0.834
Known CAD	6 (37.5)	22 (20.6)	0.340
Chronic kidney disease	2 (12.5)	12 (11.2)	0.880
**Echocardiography**			
Left ventricular function, %, mean (± SD)	49 (± 12)	58 (± 6)	**0.034**
Left ventricular hypertrophy, *n* (%)	10 (90.9)	59 (67.8)	0.162
Visually estimated normal right ventricular function, *n* (%)	6 (54.5)	75 (86.2)	**0.001**
Visually estimated impaired right ventricular function, *n* (%)	5 (45.5)	8 (9.2)	**0.001**
Right ventricular dilatation, *n* (%)	5 (45.5)	41 (47.1)	0.762
TAPSE, mm, mean (± SD)	21 (± 6)	23 (± 5)	0.397
TAPSE < 20 mm, *n* (%)	4 (36.4)	4 (4.6)	0.076
RV-FAC (%)	30 (± 10.0)	38 (± 8.5)	**0.008**
RV pressure, mmHg, mean (± SD)	30 (± 11)	29 (± 11)	0.712
Aortic stenosis > 1, *n* (%)	1 (9.1)	3 (3.4)	0.388
Aortic regurgitation > 1, *n* (%)	0 (0.0)	10 (11.5)	0.431
Mitral regurgitation > 1, *n* (%)	3 (27.3)	21 (24.1)	0.495
Tricuspid regurgitation > 1, *n* (%)	7 (63.6)	23 (23.4)	**0.018**
Pericardial effusion, *n* (%)	4 (36.4)	41 (47.1)	0.520
**Electrocardiography**			
Rate, bpm, mean (± SD)	93 (± 25)	84 (± 22)	0.268
Sinus rhythm, *n* (%)	9 (75)	71 (81.6)	0.476
QRS, ms, mean (± SD)	101 (± 14)	93 (± 22)	0.134
Regular *R* progression, *n* (%)	4 (33.3)	50 (57.5)	0.065
Right bundle branch block, *n* (%)	1 (8.3)	3 (5.3)	0.606
Left bundle branch block, *n* (%)	0 (0.0)	1 (1.1)	0.704
PQ segment, ms, mean (± SD)	155 (± 24)	174 (± 99)	0.175
QTc, ms, mean (± SD)	451 (± 33)	444 (± 34)	0.457
Negative *T* wave, *n* (%)	1 (8.3)	12 (21.1)	0.896
ST segment depression, *n* (%)	0 (0.0)	1 (1.8)	0.896
ST segment elevation, *n* (%)	0 (0.0)	0 (0.0)	0.668
**Laboratory values at admission, median (25th/75th percentile)**			
Leucocytes, 1000/µl	8.5 (6.6/1.3)	6.3 (4.3/8.7)	**0.016**
Lymphocytes, 1000/µl	0.7 (0.4/1.2)	0.8 (0.6/1.1)	0.428
Creatinin, mg/dl	1.1 (0.8/2.3)	0.9 (0.7/1.3)	0.260
GFR, ml/m^2^	69 (22/87)	74 (51/91)	0.321
d-dimers, µg/dl	2.6 (1.2/21.0)	1.1 (0.6/2.7)	**0.003**
C-reactive protein, mg/dl	19.9 (10.9/30.0)	6.7 (2.3/14.6)	**0.001**
Procalcitonin, ng/ml	0.8 (0.1/2.6)	0.1 (0.1/0.5)	**0.002**
Troponin I, ng/dl	24 (16/120)	14 (5/29)	**0.023**
NT pro-BNP, ng/l	1992 (416/7719)	377 (132/1914)	**0.041**
CK, U/l	485 (295/1332)	124 (73/258)	**< 0.001**
AST, U/l	89 (54/136)	39 (24/56)	**< 0.001**
ALT, U/l	47 (19/84)	32 (21/46)	0.115
LDH, U/l	478 (380/547)	311 (229/414)	**0.001**
**Medication at admission,** ***n*** **(%)**			
Oral anticoagulation	0 (0.0)	15 (14.0)	0.138
ACEi/ARB	8 (50.0)	52 (48.6)	0.433
Aldosterone inhibitors	4 (25.0)	11 (10.3)	**0.041**
Diuretics	5 (31.2)	35 (32.7)	0.745
Calcium channel blockers	3 (18.8)	23 (21.5)	0.952
Beta blockers	6 (37.5)	37 (34.6)	0.462
Statins	3 (18.8)	38 (35.5)	0.314
ASA	3 (18.8)	24 (22.4)	0.984
P2Y12 inhibitors	0 (0)	3 (2.8)	0.532

A *p*-value <0.05 was considered as statistically significant

**Fig. 1:**
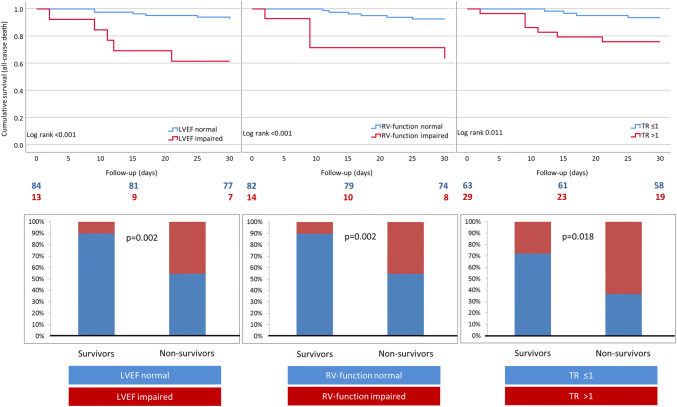
Upper row: Kaplan–Meier curves showing cumulative event-free survival for the endpoint all-cause death stratified according to LVEF, RV function and TR. Lower row: bar diagrams showing distribution of LVEF, RV-function and TR between survivors and non-survivors. *TR* tricuspid regurgitation

The overall 30-day mortality of our hospitalized patients was 13% (*n* = 16). The majority of deceased patients received ventilation therapy for acute or progressive pulmonary failure and developed multiorgan failure refractory to intensive care treatment (*n* = 14). 56 patients required ICU treatment, 49 patients mechanical ventilation, and 6 patients ECMO therapy. 6 patients were transiently treated for acute cardiovascular complains on the IMC-chest pain unit, and 61 patients were treated on a regular ward. 22 patients were dialyzed during hospital stay. Mean hospital stay was 7.4 days (± 5.4), whereas in non-survivors, days from admission to death were 10.2 (± 7.5). Patients with impaired LVEF, impaired RV-function, and a tricuspid regurgitation > 1 had a significantly higher mortality than patients with normal LVEF, normal RV-function, and mild tricuspid regurgitation ≤ 1 (Fig. 1). Patients with impaired LVEF and RV-function showed a significantly worse cumulative event-free survival compared to patients with normal LVEF and RV-function [log rank < 0.001 and log rank < 0.001 for all-cause death, respectively (Fig. 1)]. Furthermore, tricuspid regurgitation > 1 was associated with a significantly worse cumulative event-free survival [log rank 0.011 for all-cause death (Fig. 1)].

**Fig. 2:**
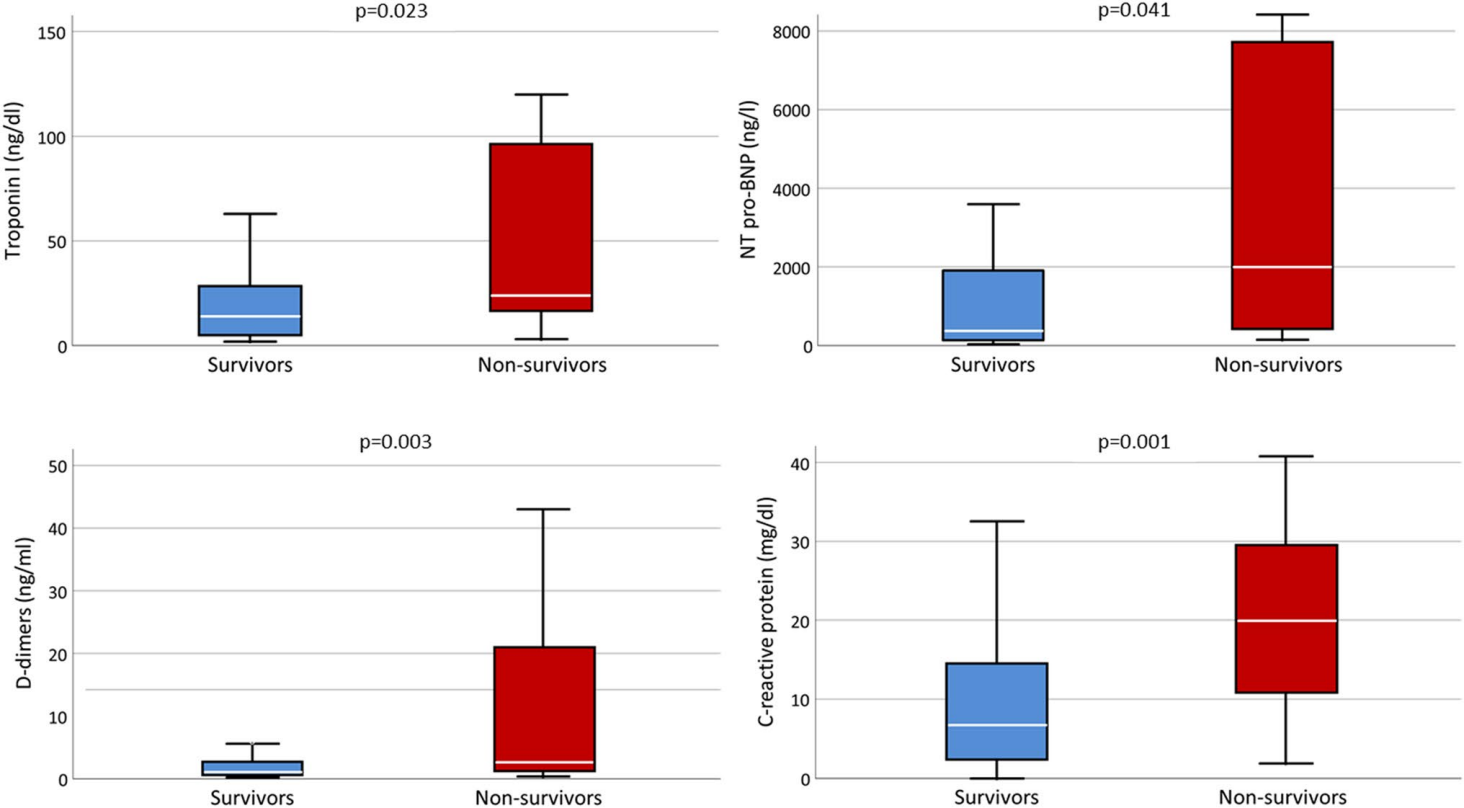
Laboratory values at admission stratified according to survivors and non-survivors

Moreover, in non-survivors leucocyte count, d-dimers, C-reactive protein, procalcitonin, troponin-I, NT pro-BNP, CK, AST, and LDH levels were significantly higher and treatment with aldosterone antagonists was significantly more frequent when compared to survivors (Table 2, Fig. 2). LVEF at admission did not correlate with D-dimers (rho = − 0.155, *p* = 0.116). LVEF at admission was, however, significantly associated with troponin I and NT pro-BNP (rho = − 0.367, *p* < 0.001 and rho = − 0.485, *p* < 0.001, respectively). Furthermore, RV-FAC at admission did not significantly correlate with d-dimers (rho = − 0.103, *p* = 0.321) but was significantly associated with troponin I and NT pro-BNP (rho = − 0.442, *p* < 0.001 and rho = − 0.304, *p* = 0.006, respectively). Cumulative event-free survival was lower by trend in patients with reduced RV-FAC when compared to those with normal RV-FAC (Log rank = 0.280 for RV-FAC cut off 35% and Log rank = 0.056 for RV-FAC cut off 30%, respectively).

Cox-regression analysis with mortality as dependent and age, arterial hypertension, diabetes mellitus type 2, LVEF, RV-function, and tricuspid regurgitation > 1 as independent variables revealed that besides diabetes mellitus, LVEF was significantly and independently associated with all-cause mortality (Table 3). Due to the low event rate multivariate analysis warrants, however, cautious interpretation.

**Table 3:** Cox-regression analysis with forward variable selection with mortality as dependent and age, arterial hypertension, diabetes mellitus type 2, LVEF, RV-function, and tricuspid regurgitation > 1 as independent variables

Variable	HR (95% CI)	*p* value
Diabetes mellitus type 2	3.65 (1.06–12.63)	**0.041**
LVEF	12.19 (2.87–51.83)	**0.001**

## Discussion

The main findings of the present study are: (i) in a consecutive collective of symptomatic COVID-19-positive patients with respiratory distress, impaired systolic left and right ventricular function as well as relevant tricuspid regurgitation are associated with 30-day all-cause mortality. (ii) Elevated levels of myocardial distress markers (troponin-I, NT pro-BNP) are associated with poor prognosis in COVID-19 patients.

Our findings in line with previous reports confirm that cardiac injury is a major concern and occurs frequently in COVID-19 patients with respiratory failure. Severe respiratory distress has been considered to be the main cause of COVID-19-associated deaths. Recently, Shi et al. have found that 7.2% of hospitalized COVID-19 patients develop cardiac injury, which increases up to approximately 20% when patients are referred to intensive care units [21]. Patients with pre-existing cardiovascular disease seem to be more susceptible to COVID-19 than those lacking this condition [22].

At present, the pathophysiological mechanisms of COVID-19 and cardiac injury and heart failure are poorly understood. However, it seems likely as known for other infectious diseases that COVID-19-induced systemic inflammatory responses may contribute to myocardial failure especially in patients with known heart disease [23, 24]. Further, our findings indicate that right ventricular stress as indicated by the presence of the right ventricular failure and a relevant tricuspid regurgitation might be a consequence of COVID-19-induced pulmonary distress with development of elevation of the pulmonary artery pressure [25]. This explanation is further strengthened by the observation that the majority of severely affected COVID-19 patients reveal elevated levels of NT pro-BNP levels indicative of acute myocardial stress [26]. We could, however, not show significant associations between elevated pulmonary artery pressure and mortality in COVID-19 patients. In the present study, we applied conventional echocardiographic diagnosis without performing LV and RV strain analyses, which may be more adequate to detect subtle changes in myocardial function. However, at the time being, we were not able to analyse the echocardiographic strain for this first COVID-19 wave at our institution due to logistical challenges.

At present, we do not have a causal therapy for COVID-19 affected patients. However, our data and an increasing number of reports strongly suggest that the thorough assessment of cardiac function is an absolute requirement in COVID-19 patient care. After the published statement of the German Cardiac Society in March 2020, we continued and initiated guideline-recommended HF therapy in every patient with impaired LV- and/or RV-function as well as elevated NT pro-BNP plasma levels. The type of medical HF therapy included ARBs, ACE, aldosterone antagonists and ß-Blockers. None of the 123 patients received Sacubitril/Valsartan. A rigorous treatment of cardiac dysfunction according to the well-established and recommended international guidelines should be a cornerstone for patients with COVID-19. Turning away from effective treatment options may do further harm to affected COVID-19 patients. On the other hand, following our cardiology guidelines is at present one of the few possibilities to save lives. Thus, a formation of a competent Cardio-COVID-19 team is one major critical measure in fighting the threatening disease.

## Conclusions

Cardiac failure is associated with poor prognosis in patients COVID-19 infection. A comprehensive assessment of cardiac function is required in patients care with symptoms of acute COVID-19 infection.

## Data Availability

Not applicable.

## References

[1] Mahase E (2020). Coronavirus covid-19 has killed more people than SARS and MERS combined, despite lower case fatality rate. BMJ.

[2] Zheng YY, Ma YT, Zhang JY, Xie X (2020). COVID-19 and the cardiovascular system. Nat Rev Cardiol.

[3] Bajwa EK, Boyce PD, Januzzi JL, Gong MN, Thompson BT, Christiani DC (2007). Biomarker evidence of myocardial cell injury is associated with mortality in acute respiratory distress syndrome. Crit Care Med.

[4] Li G, Li H, Lu J (2020). No adequate evidence indicating hypertension as an independent risk factor for COVID-19 severity. Clin Res Cardiol.

[5] Li B, Yang J, Zhao F, Zhi L, Wang X, Liu L, Bi Z, Zhao Y (2020). Prevalence and impact of cardiovascular metabolic diseases on COVID-19 in China. Clin Res Cardiol.

[6] Kobayashi M, Girerd N, Duarte K, Preud'homme G, Pitt B, Rossignol P (2020). Prognostic impact of plasma volume estimated from hemoglobin and hematocrit in heart failure with preserved ejection fraction. Clin Res Cardiol.

[7] European Society of Cardiology (2020) ESC guidance for the diagnosis and management of CV disease during the COVID-19 pandemic. https://www.escardio.org/Education/COVID-19-and-Cardiology/ESC-COVID-19-Guidance. Accessed 30 Apr 2020

[8] Oren OKS, Gluckman TJ, Gersh BJ, Blumenthal RS (2020) Coronavirus disease 2019 (COVID-19): epidemiology, clinical spectrum and implications for the cardiovascular clinician. https://www.acc.org/latest-in-cardiology/articles/2020/04/06/11/08/covid-19-epidemiology-clinical-spectrum-and-implications-for-the-cv-clinician. Accessed 30 Apr 2020

[9] Lakkireddy DR, Chung MK, Gopinathannair R, Patton KK, Gluckman TJ, Turagam M, Cheung J, Patel P, Sotomonte J, Lampert R, Han JK, Rajagopalan B, Eckhardt L, Joglar J, Sandau K, Olshansky B, Wan E, Noseworthy PA, Leal M, Kaufman E, Gutierrez A, Marine JM, Wang PJ, Russo AM (2020). Guidance for cardiac electrophysiology during the coronavirus (COVID-19) pandemic from the Heart Rhythm Society COVID-19 Task Force; Electrophysiology Section of the American College of Cardiology; and the Electrocardiography and Arrhythmias Committee of the Council on Clinical Cardiology, American Heart Association. Heart Rhythm.

[10] Inciardi RM, Lupi L, Zaccone G, Italia L, Raffo M, Tomasoni D, Cani DS, Cerini M, Farina D, Gavazzi E, Maroldi R, Adamo M, Ammirati E, Sinagra G, Lombardi CM, Metra M (2020). Cardiac involvement in a patient with coronavirus disease 2019 (COVID-19). JAMA Cardiol.

[11] Lippi G, Lavie CJ, Sanchis-Gomar F (2020). Cardiac troponin I in patients with coronavirus disease 2019 (COVID-19): Evidence from a meta-analysis. Prog Cardiovasc Dis.

[12] World Medical Association Declaration of Helsinki (1997). Recommendations guiding physicians in biomedical research involving human subjects. Cardiovasc Res.

[13] ICH Harmonised Tripartite Guideline: Guideline for Good Clinical Practice (2001). J Postgrad Med 47 (3):199–203 11832625

[14] Directive 2001/20/EC of the European Parliament and of the Council of 4 April 2001 on the approximation of the laws, regulations and administrative provisions of the member states relating to the implementation of good clinical practice in the conduct of clinical trials on medicinal products for human use (2002). Med Etika Bioet 9(1–2):12–19 16276663

[15] Schiller NB, Shah PM, Crawford M, DeMaria A, Devereux R, Feigenbaum H, Gutgesell H, Reichek N, Sahn D, Schnittger I (1989). Recommendations for quantitation of the left ventricle by two-dimensional echocardiography. American Society of Echocardiography Committee on Standards, Subcommittee on Quantitation of Two-Dimensional Echocardiograms. J Am Soc Echocardiogr.

[16] Lang RM, Badano LP, Mor-Avi V, Afilalo J, Armstrong A, Ernande L, Flachskampf FA, Foster E, Goldstein SA, Kuznetsova T, Lancellotti P, Muraru D, Picard MH, Rietzschel ER, Rudski L, Spencer KT, Tsang W, Voigt JU (2015). Recommendations for cardiac chamber quantification by echocardiography in adults: an update from the American Society of Echocardiography and the European Association of Cardiovascular Imaging. J Am Soc Echocardiogr.

[17] Lancellotti P, Moura L, Pierard LA, Agricola E, Popescu BA, Tribouilloy C, Hagendorff A, Monin JL, Badano L, Zamorano JL, European Association of E (2010). European Association of Echocardiography recommendations for the assessment of valvular regurgitation. Part 2: mitral and tricuspid regurgitation (native valve disease). Eur J Echocardiogr.

[18] Nishimura RA, Otto CM, Bonow RO, Carabello BA, Erwin JP, Guyton RA, O'Gara PT, Ruiz CE, Skubas NJ, Sorajja P, Sundt TM, Thomas JD, American College of Cardiology/American Heart Association Task Force on Practice G (2014). 2014 AHA/ACC guideline for the management of patients with valvular heart disease: executive summary: a report of the American College of Cardiology/American Heart Association Task Force on Practice Guidelines. J Am Coll Cardiol.

[19] Lancellotti P, Tribouilloy C, Hagendorff A, Moura L, Popescu BA, Agricola E, Monin JL, Pierard LA, Badano L, Zamorano JL, European Association of E (2010). European Association of Echocardiography recommendations for the assessment of valvular regurgitation. Part 1: aortic and pulmonary regurgitation (native valve disease). Eur J Echocardiogr.

[20] Parasuraman S, Walker S, Loudon BL, Gollop ND, Wilson AM, Lowery C, Frenneaux MP (2016). Assessment of pulmonary artery pressure by echocardiography—a comprehensive review. Int J Cardiol Heart Vasc.

[21] Shi S, Qin M, Shen B, Cai Y, Liu T, Yang F, Gong W, Liu X, Liang J, Zhao Q, Huang H, Yang B, Huang C (2020). Association of cardiac injury with mortality in hospitalized patients with COVID-19 in Wuhan, China. JAMA Cardiol.

[22] Yang J, Zheng Y, Gou X, Pu K, Chen Z, Guo Q, Ji R, Wang H, Wang Y, Zhou Y (2020). Prevalence of comorbidities in the novel Wuhan coronavirus (COVID-19) infection: a systematic review and meta-analysis. Int J Infect Dis.

[23] Barnett R (2019). Influenza. Lancet.

[24] Ikonomidis I, Pavlidis G, Katsimbri P, Andreadou I, Triantafyllidi H, Tsoumani M, Varoudi M, Vlastos D, Makavos G, Kostelli G, Betaenas D, Lekakis J, Parissis J, Boumpas D, Alexopoulos D, Iliodromitis E (2019). Differential effects of inhibition of interleukin 1 and 6 on myocardial, coronary and vascular function. Clin Res Cardiol.

[25] Solaimanzadeh I (2020). Acetazolamide, nifedipine and phosphodiesterase inhibitors: rationale for their utilization as adjunctive countermeasures in the treatment of coronavirus disease 2019 (COVID-19). Cureus.

[26] Deng Q, Hu B, Zhang Y, Wang H, Zhou X, Hu W, Cheng Y, Yan J, Ping H, Zhou Q (2020). Suspected myocardial injury in patients with COVID-19: evidence from front-line clinical observation in Wuhan, China. Int J Cardiol.

